# Rice Mitogen Activated Protein Kinase Kinase and Mitogen Activated Protein Kinase Interaction Network Revealed by *In-Silico* Docking and Yeast Two-Hybrid Approaches

**DOI:** 10.1371/journal.pone.0065011

**Published:** 2013-05-30

**Authors:** Dhammaprakash Pandhari Wankhede, Mohit Misra, Pallavi Singh, Alok Krishna Sinha

**Affiliations:** National Institute of Plant Genome Research, New Delhi, India; University of Toronto, Canada

## Abstract

Protein-protein interaction is one of the crucial ways to decipher the functions of proteins and to understand their role in complex pathways at cellular level. Such a protein-protein interaction network in many crop plants remains poorly defined owing largely to the involvement of high costs, requirement for state of the art laboratory, time and labour intensive techniques. Here, we employed computational docking using ZDOCK and RDOCK programmes to identify interaction network between members of *Oryza sativa* mitogen activated protein kinase kinase (MAPKK) and mitogen activated protein kinase (MAPK). The 3-dimentional (3-D) structures of five MAPKKs and eleven MAPKs were determined by homology modelling and were further used as input for docking studies. With the help of the results obtained from ZDOCK and RDOCK programmes, top six possible interacting MAPK proteins were predicted for each MAPKK. In order to assess the reliability of the computational prediction, yeast two-hybrid (Y2H) analyses were performed using rice MAPKKs and MAPKs. A direct comparison of Y2H assay and computational prediction of protein interaction was made. With the exception of one, all the other MAPKK-MAPK pairs identified by Y2H screens were among the top predictions by computational dockings. Although, not all the predicted interacting partners could show interaction in Y2H, yet, the harmony between the two approaches suggests that the computational predictions in the present work are reliable. Moreover, the present Y2H analyses *per se* provide interaction network among MAPKKs and MAPKs which would shed more light on MAPK signalling network in rice.

## Introduction

The genetic potential of improved crop cultivars to perform better in field conditions is often constrained by a wide spectrum of biotic and abiotic stresses. These factors directly and indirectly affect the growth and development of plants and thus ultimately result in lower production of food grains. In order to develop crop cultivars resistant/tolerant to an array of stresses, it is a prerequisite to understand how plants perceive and transduce the cues and generate responses to cope up with the stress in question. Protein kinases are crucial players, not only in regulating various stress responses but also in growth and development. Among the protein kinases, the highly conserved mitogen-activated protein kinase (MAPK) pathways in eukaryotes play pivotal role in basic cellular process, development, hormone biosynthesis/signalling, senescence, plant immunity as well as in producing responses to several stress conditions [1]–[3]. Much of our knowledge comes from the model plant *Arabidopsis thaliana* while in the case of most of the crop plants the knowledge about these facets is still in its nascent stage.

The MAPK cascades are composed of three main components, MAPKKK (MKKK/MEKK), MAPKK (MKK/MEK) and MAPK (MPK) which are activated through consecutive phosphorylations. Arabidopsis genome comprises 60–80 MAPKKK, 10 MAPKK and 20 MAPK genes [4], [5]. On the basis of rice genome sequence, the members of rice MAPK cascade have been identified, which comprise 75 putative MAPKKKs, 8 MAPKKs and 15 MAPKs [5], [6]. Later, an additional MAPK was identified in our laboratory (OsMPK16-2, Acc No. EU675865) thus making the number of MAPK to be 16 in indica cultivar of rice. However, in order to understand MAPK signalling completely, it is important to understand the interaction networks between the members of MAPK cascade.

The available literature showing activation of downstream kinases by its upstream kinase module in response to specific stimuli seems to be quite complex as well as redundant. Discrepancy in the number of MAPKK and MAPK genes suggests that a single MAPKK is likely to activate multiple MAPKs. Further, an individual MAPK protein may serve as a target for multiple upstream MAPKK [3], [7]–[10]. Although the interactions between the components of MAPK cascade have been well studied in *A. thaliana,* there is lack of such studies in crop plants. Surpisingly in rice very few MAPKKs-MAPKs interactions have been identified either through yeast two-hybrid (Y2H) or phosphorylation assays [11]–[14]. Comprehensive Y2H analysis of several rice protein kinases has also revealed only a few MAPKK-MAPK interactions [12]. Recently Singh et al. [15] have reported rice MAPK interactome analysis using directed as well as proteome wide protein-protein interactions. Although, the study has presented a comprehensive interaction network between rice MAPKK, a few MAPKs and transcription factors, yet it throws limited light on MAPKK-MAPK aspects of interaction network and showed protein-protein interactions of only four of the fifteen MAPKs. Therefore, it is important to study the interactions among the components of the MAPK cascade which would form a pivotal in signalling and regulatory controls as well as in machinery of cellular function.

The advancements in the field of bioinformatics have given us efficient tools to understand several biological processes at molecular level. In recent times a significant progress has been made in computational modelling of protein structures and molecular docking, which holds a great promise in prediction of protein-protein interactions. Docking is the computational scheme that attempts to find the best matching between two molecules: a receptor and ligand [16]. Protein-protein docking is one of the potential means to study the structure of protein-protein complexes such as antibody-antigen complexes [17], [18], [19]. Similar methodology can be used to study if a given protein has a potential to interact with itself. However, the availability of the individual protein structures as either X-ray crystal structures or structures determined through nuclear magnetic resonance (NMR) is always a prerequsite for such studies. Homology models designed using high sequence similarity template can also be used in the docking studies [20].

In the present work, homology modelling approach was employed to determine 3D structure of rice MAPKKs and MAPKs. These 3D structures were further used as an input for protein-protein docking using ZDOCK and RDOCK programmes, to predict MAPKK-MAPK interactions. Simultaneously, Y2H analyses were used to study rice MAPKK-MAPK protein-protein interaction networks. A direct comparison of computational prediction and Y2H analyses of MAPKK and MAPK was made to assess the reliability of computational docking for prediction of protein-protein interactions.

## Materials and Methods

### *In-silico* Homology Modelling

Homology modelling was performed as mentioned [21]. For selection of templates for homology modelling of selected proteins, PSI BLAST [22] was performed against the PDB database (http://www.pdb.org/pdb/home/home.do) [23]. Only the hits with >30% sequence identity were selected. Proteins used as templates for homology modelling, along with PDB IDs and their identity with the target proteins are shown in Table S1. Discovery studio 2.5.5.9350 (http://www.accelrys.com/dstdio) suite was used, which is a ClustalW hybrid for sequence alignment and modeler [24], for the homology model building. 3D Models were refined with the help of loop refinement (MODELER and looper algorithm based) and side chain refinement protocols. Evaluation of 3D models was done by drawing Ramachandran plot and running the verify protein (Profiles 3D) protocol. Prepare protein protocol was finally run on protein models. The protocol executes the following steps, (i) cleans the protein, (ii) optimizes side-chain conformation for residues with inserted atoms, (iii) removes water molecules (optional), (iv) breaks bonds between metal and protein atoms (optional), (v) models missing loop regions based on SEQRES information or by user-definition (optional).

### Protein–protein Docking

For protein–protein docking, the ZDOCK and RDOCK programs were used as mentioned in [21]. ZDOCK is a rigid body protein docking algorithm that explicitly searches rotational space and uses a Fast Fourier Transformation (FFT) algorithm, to significantly speed up searching in translational spaces [25]. ZDOCK score is the shape complementarity score calculated by the ZDOCK program [26]. ZRank score is the energy of the docked posed calculated by the ZRank rescoring method. The Process Poses (ZDOCK) protocol allows the selection of a subset from a set of docked protein poses, generated by the Dock Proteins (ZDOCK) protocol, either according to pose rank or by specifying residues on the docking interface. RDOCK program is an energy minimization algorithm [27], designed as refinement re-ranking tool for ZDOCK’s top predictions. In initial stage the protein receptor (MAPKs) and protein ligand (MAPKKs) were treated as rigid bodies and all rotational and translational degrees of freedom were fully explored, with the scoring functions that were tolerant to conformational changes. An angular step of 15° was used which resulted in 2600 poses. In the refinement stage, RDOCK top poses of near native structure obtained in the initial stage were refined and reranked. RDOCK minimization of the complexes generated by ZDOCK comprised small clashes removal to allow small conformational changes, optimization of polar interactions and charged interactions.

### Yeast Two Hybrid Assay for One to One Protein Interaction

In our laboratory, all the sixteen rice *MAPKs* and five of the six functional *MAPKKs* have already been cloned from the Pusa Basmati 1 cultivar of indica rice and these sequences are available in GenBank database [28]. Plasmids from these clones were used as templates for PCR amplification of *OsMPKs* and *OsMKKs*, using gene specific primers which had specific restriction enzyme recognition sequence as an adapter. Fourteen rice *MAPKs*, *OsMPK3* (DQ826422), *OsMPK4* (FJ621301), *OsMPK6* (FJ621301), *OsMPK7* (DQ826424), *OsMPK14* (EU675864), *OsMPK16-1* (EU779804), *OsMPK16-2* (EU675865), *OsMPK17-1* (EU675866), *OsMPK17-2* (DQ826423), *OsMPK20-1* (DQ826423), *OsMPK20-2* (FJ907414), *OsMPK20-3* (EU675869), *OsMPK20-4* (DQ826425), *OsMPK20-5* (EU675870), *OsMPK21-2* (FJ621303) and five *MAPKKs*, *OsMKK1* (EF529623.1), *OsMKK3* (EF392366), *OsMKK4* (JQ886088), *OsMKK6* (DQ779790.1) and *OsMKK10-2* (EF666056.1) were cloned in pGADT7 and pGBKT7 vectors (BD Biosciences). Rice MAPKs nomeclature accroding to Hamel et al. [5] has been followed. These clones were then used for Y2H screening to study OsMAPKK and OsMAPKs interactions. A Matchmaker yeast two-hybrid system (BD bioscience, USA) was used to check protein-protein interactions. For yeast transformation, yeast competent cells (AH109) were prepared according to manufacturer’s instructions. OsMAPKs and OsMAPKKs constructs were co-transformed in AH109 competent cells. Co transformants were initially selected on nutrient medium lacking Leu and Trp (SD/−Leu/−Trp). The resultant co-transformed cells were then streaked on drop-out medium deficient in Ade, His Leu and Trp (SD/−Ade/−His/−Leu/−Trp).

## Results

### Homology Modelling of OsMAPKKs and OsMAPKs

In order to understand the molecular interaction properties of a protein, it is a prerequisite to have the information about its 3D structure. However, in the absence of crystallographic structures for rice MAPKs and MAPKK, the homology modelling approach was employed to determine a reasonable 3D structure of these proteins based on the known structure of the template proteins. To select the template for homology modelling Psi Blast [22] was performed for rice MAPKK and MAPK proteins against the PDB database (Protein Data Bank) [23] in order to obtain even the remote homologs of target proteins. Homologs only with >30% sequence similarity were selected. Unfortunately owing to unavailability of suitable templates, five MAPKs (OsMPK16-2, OsMPK17-2, OsMPK20-1, OsMPK20-4 and OsMPK21-1) and three MAPKKs (OsMKK1, OsMKK10-1 and OsMKK10-3) were not considered for homology modelling. The proteins used as templates for homology modelling along with PDB identities (IDs) are shown in Table S1. In order to have precise 3D model of target protein, multiple templates were used so that the entire length of the proteins was covered. The template proteins were aligned through modeller. To build the homology models Modeler 9v8 [24] was employed. Thus using Discovery studio 2.5.5.9350 (http://www.accelrys.com/dstdio) suite, 3D structure of twelve rice MAPKs and five MAPKKs were determined.

**Figure 1:**
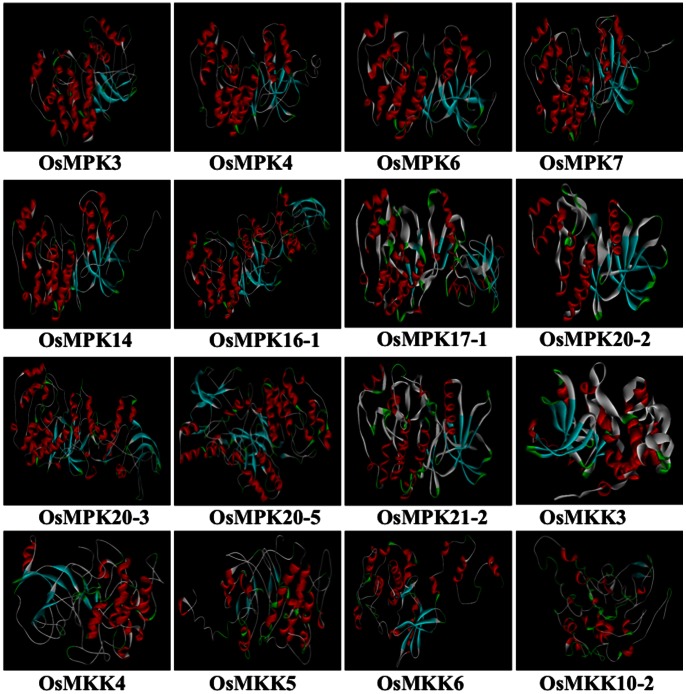
Theoretical 3D models of rice MAPKKs and MAPKs build by homology modelling . Structure of eleven rice MAP kinases (OsMPK3, OsMPK4, OsMPK6, OsMPK7, OsMPK14, OsMPK16-1, OsMPK17-1, OsMPK20-2, OsMPK20-3, OsMPK20-5 and OsMPK21-2) and five MAP kinase kinases (OsMKK3, OsMKK4, OsMKK5, OsMKK6, OsMKK10-2) are shown. The red region represents the alpha helices, sky blue regions the beta sheets, green coloured regions depict the turns whereas the grey colour represents the loops.

All the 3D models were refined with the help of loop refinement (MODELER and looper algorithm based) and side chain refinement protocols. The modelled 3D structure of each of the eleven MAPKs and five MAPKKs have been shown (Figure 1). The DOPE (Discrete Optimized Protein Energy) and PDF (Probability Density Function) total score for all the 3D structure are given in Table 1. DOPE score is an atomic based statistical potential in MODELER package for model evaluation and structure prediction. The DOPE score of a protein can be viewed as a conformational energy which measures the relative stability of a conformation with respect to other conformations of the same protein. The PDF energy is useful for evaluating the relative overall condition of each model. As per the protocol, lower values of DOPE score and PDF total energy represent a better model.

**Table 1:** Verification of 3D structures of rice MAPKs and MAPKKs.

Structure	DOPE Score	PDF total Score	Verify Expected low Score	Verify Expected high Score	Verify Score
OsMPK3	−39379.9	16306.1	75.6846	168.188	**114.02**
OsMPK 4	−42867.3	−11614	77.132	171.404	**152.75**
OsMPK 6	−42982	−11238.3	75.4779	167.729	**132.08**
OsMPK 7	−44612.3	−11605.5	75.6846	168.188	**139.02**
OsMPK 14	−43626.9	−11183.1	74.4442	165.432	**156.87**
OsMPK 16-1	−52028	17858	111.511	247.802	**171.82**
OsMPK 17-1	−43897.8	13794.5	92.2379	204.973	**136.81**
OsMPK 20-2	−27185.8	10117.4	51.9419	115.426	**80.72**
OsMPK 20-3	−60734.3	−15050.3	117.319	260.708	**145.48**
OsMPK 20-5	−60034.2	−15250.1	121.884	270.853	**180.85**
OsMPK 21-2	−36514.3	14152.4	73.204	162.676	**128.24**
OsMKK 3	−32101.9	10256.8	65.5596	145.688	**94.31**
OsMKK 4	−26487.8	−1076.66	75.6846	168.188	**121.5**
OsMKK 5	−32387.3	−7646.87	70.1041	155.787	**108.29**
OsMKK 6	−35119.7	12470.1	72.7906	161.757	**110.54**
OsMKK 10-2	−28001	11417.4	69.4843	154.409	**70.7**

DOPE–Discrete Optimized Protein Energy, PDF–Probability Density Function.

### Model Assessment

**Figure 2:**
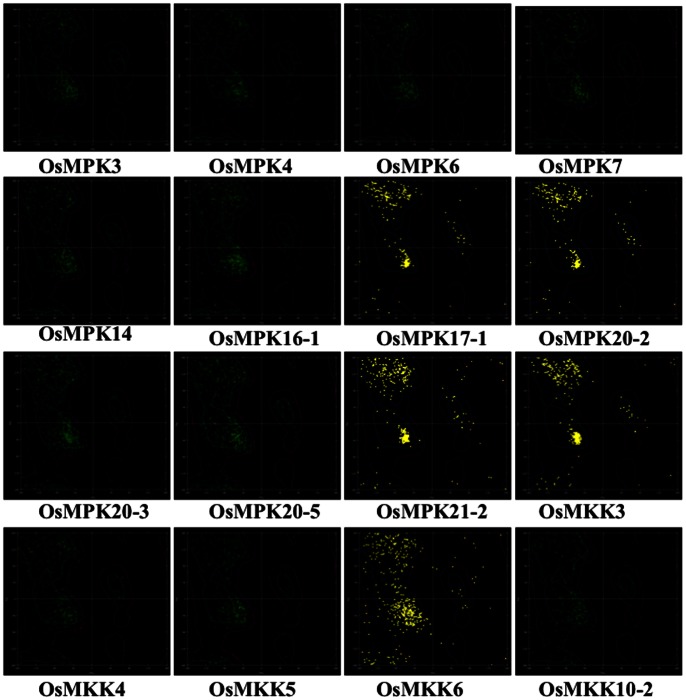
Ramachandran plot analysis of theoretical 3D structure of rice MAPKKs and MAPKs. The 3D Structures of eleven rice MAP kinases (OsMPK3, OsMPK4, OsMPK6, OsMPK7, OsMPK14, OsMPK16-1, OsMPK17-1, OsMPK20-2, OsMPK20-3, OsMPK20-5 and OsMPK21-2) and five MAP kinase kinases (OsMKK3, OsMKK4, OsMKK5, OsMKK6, OsMKK10-2) were validated using Ramachandran plot. The green dots/yellow dots show the amino acids that are in the most favoured regions and additionally allowed region while red dots show the amino acids that are in generously allowed region or disallowed regions. The regions covered by sky blue line show most favoured regions, while the regions covered by pink line show additionally allowed regions. Other regions of the plot show the generously allowed or disallowed region.

The overall stereochemical quality of the modelled 3D structure of proteins were evaluated by using Ramachandran plot which is based on psi (Cα-C bond) and phi (N-Cα bond) angles of the protein and provides information about the number of amino acid residues present in allowed and disallowed regions. All the modelled proteins showed maximum residues in the most favoured region followed by allowed region and least in the generously allowed regions in the Ramachandran plot (Figure 2). Very few residues were also found lying in the disallowed regions. In all, the results suggest the reliability of the modelled proteins.

Additionally reliability of the structures for docking studies was evaluated by employing the ‘Verify Protein protocol’ (profile 3-D method) for testing a preliminary protein structure based on experimental data. It depends on the principle, that a protein's structure must be compatible with its own sequence. The ‘Verify Protein’ was used to calculate local 3D-1D scores in a fixed length sliding window (typically about 5 to 20 residues long) and plotted against residue position. This reveals local regions of relatively high or low 3D-1D compatibility [29]. The program evaluates fitness of a protein sequence in its current 3D environment. Line plots for all the 3D structures of proteins were drawn and the values of the verify score are given in Table 1. The verification scores of all the sixteen modelled proteins lie between the low and high expected verify score indicating that the modelled protein structures are of acceptable quality. After verification, the ‘prepare protein’ protocol was run on the generated models which ensured removal of any alternate conformations.

### Protein-protein Docking for Identification of Interaction between Rice MAPKKs and MAPKs

The docking of rice MAPKKs and MAPKs was performed following ZDOCK and RDOCK programs. ZDOCK is a docking program that predicts several protein complexes using Pairwise Shape Complementarity (PSC) of input protein structure [26]. The output results of RDOCK contain two important scores, ZRANK and the E_RDOCK (Energy RDOCK). For prediction of the better docking pose ‘E_RDOCK score’ is often preferred over ZRANK score [27]. The clashes in the selected poses were zero thereby suggesting better docking positions. Higher negative score of E_RDOCK could be used to predict the possible protein-protein interactions from a set of proteins since it indicates stronger interaction.

**Figure 3:**
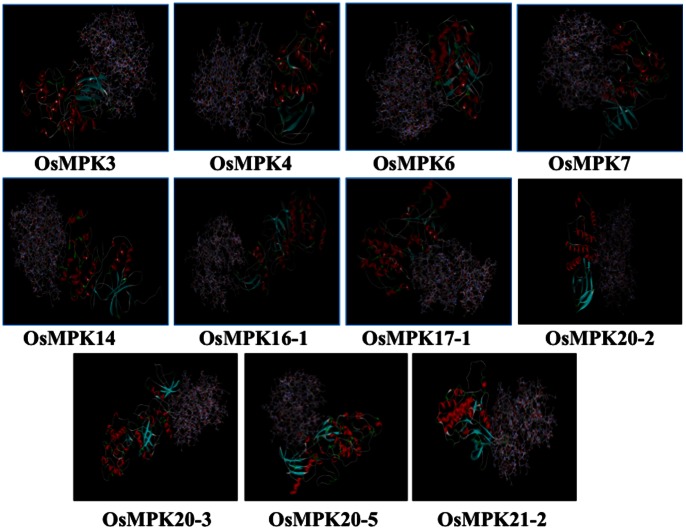
Top docking poses of OsMKK3 with rice MAPKs. The best docking positions of OsMKK3 with each of the eleven rice MAPKs (OsMPK3, OsMPK4, OsMPK6, OsMPK7, OsMPK14, OsMPK16-1, OsMPK17-1, OsMPK20-2, OsMPK20-3, OsMPK20-5 and OsMPK21-2) are shown. OsMKK3 is represented as a wireframe pattern whereas all OsMAPKs as a solid ribbon form.

The results obtained from docking studies involving OsMKK3 and eleven MAPK modules have been presented in Table 2. The representative docking positions of OsMKK3-OsMAPKs complexes have been shown in Figure 3. The lowest ‘E_RDOCK score’ was recorded for OsMKK3-OsMPK20-3 (−27.31) followed by OsMKK3-OsMPK21-2 (−27.20) while OsMKK3-OsMPK6 showed highest E_RDOCK score (−12.49). Among the eleven OsMAPKs top six putative interacting partners of OsMKK3 were OsMPK20-3, OsMPK21-2, OsMPK20-2 (−24.48), OsMPK20-5 (−24.20), OsMPK14 (−20.9), and OsMPK7 (−20.46).

**Table 2:** ZDOCK and RDOCK score of OsMKK3 against each of eleven rice MAPKs.

Protein Pair	Pose no.	ZDOCK Score	ZRANK Score	E_RDOCK Score	Clash
OsMPK20-3 & OsMKK3	671	14.98	−26.10	−**27.31**	0
OsMPK21-2 & OsMKK3	1	14.1	−116.38	−**27.20**	0
OsMPK20-2 & OsMKK3	815	13.30	−19.67	−**24.48**	0
OsMPK20-5 & OsMKK3	9	16.48	−30.96	−**24.20**	0
OsMPK14 & OsMKK3	162	13.46	−55.36	−**20.9**	0
OsMPK7 & OsMKK3	29	13.52	−75.1	−**20.46**	0
OsMPK3 & OsMKK3	211	14.86	−48.21	−19.42	0
OsMPK4 & OsMKK3	11	14.26	−88.23	−19.26	0
OsMPK16-1 & OsMKK3	110	15.62	−63.91	−18.72	0
OsMPK17-1 & OsMKK3	143	15.04	−57.92	−17.3	0
OsMPK6 & OsMKK3	48	18.08	−70.21	−12.49	0

E_R – Energy RDOCK score. Lower values of ZRANK score and E_RDOCK and higher ZDOCK score indicate top/better docking of the complex. Clash ‘0′ indicates no stearic clash between the proteins after refinement by RDOCK protocol.

Docking of OsMKK4 with eleven MAPKs was performed and output values are presented in Table 3. Figure S1 shows the docking positions of OsMKK4-OsMAPKs complex. Based on E_RDOCK score top six putative OsMKK4 interacting OsMPKs were OsMPK20-3 (−31.76), OsMPK21-2 (−30.56), OsMPK6 (−29.41), OsMPK20-5 (−27.28), OsMPK3 (−25.19) and OsMPK16-1 (−25.14).

**Table 3:** ZDOCK and RDOCK score of OsMKK4 against each of eleven rice MAPKs.

Protein Pair	Pose no.	ZDOCK Score	ZRANK Score	E_RDOCK Score	Clash
OsMPK20-3 & OsMKK4	527	16.64	−40.87	−**31.76**	0
OsMPK21-2 & OsMKK4	20	14.88	−86.01	−**30.56**	0
OsMPK6 & OsMKK4	7	14.28	−93.71	−**29.41**	0
OsMPK20-5 & OsMKK4	335	14.08	−49.8	−**27.28**	0
OsMPK3 & OsMKK4	4	15.26	−99.42	−**25.19**	0
OsMPK16-1 & OsMKK4	5	15.06	−95.51	−**25.14**	0
OsMPK7 & OsMKK4	51	12.9	−76.11	−22.62	0
OsMPK20-2 & OsMKK4	69	13.70	−73.82	−22.43	0
OsMPK17-1 & OsMKK4	25	14.22	−83.85	−21.92	0
OsMPK4 & OsMKK4	17	14.68	−82.4	−18.53	0
OsMPK14 & OsMKK4	30	12.8	−82.92	−17.77	0

E_R – Energy RDOCK score. Lower values of ZRANK score and E_RDOCK and higher ZDOCK score indicate top/better docking of the complex. Clash ‘0′ indicates no stearic clash between the proteins after refinement by RDOCK protocol.

For OsMKK5, top six interacting MAPKs were OsMPK20-5, OsMPK3, OsMPK17-1, OsMPK21-2, OsMPK20-3 and OsMPK6 (Table 4). The best docking positions of OsMKK5-OsMAPKs are shown in Figure S2.

**Table 4:** ZDOCK and RDOCK score of OsMKK5 against each of eleven rice MAPKs.

Protein Pair	Pose no.	ZDOCK Score	ZRANK Score	E_RDOCK Score	Clash
OsMPK20-5 & OsMKK5	242	16.7	−55.46	−**42.92**	0
OsMPK3 & OsMKK5	12	17.28	−91.13	−**33.76**	0
OsMPK17-1 & OsMKK5	50	15.4	−77.74	−**31.95**	0
OsMPK21-2 & OsMKK5	410	15.2	−44.41	−**31.68**	0
OsMPK20-3 & OsMKK5	293	15.92	−52.2	−**30.85**	0
OsMPK6 & OsMKK5	129	16.24	−65.48	−**29.35**	0
OsMPK16-1 & OsMKK5	151	15.06	−62.35	−29.24	0
OsMPK20-2 & OsMKK5	235	12.8	−54.96	−29.05	0
OsMPK7 & OsMKK5	78	13.86	−72.64	−25.17	0
OsMPK14 & OsMKK5	27	13.44	−85.27	−20.8	0
OsMPK4 & OsMKK5	32	16.2	−78.96	−20.61	0

E_R – Energy RDOCK score. Lower values of ZRANK score and E_RDOCK and higher ZDOCK score indicate top/better docking of the complex. Clash ‘0′ indicates no stearic clash between the proteins after refinement by RDOCK protocol.

Top six predictions of OsMKK6 interacting OsMAPKs were OsMPK20-2, OsMPK16-1, OsMPK21-2, OsMPK20-5, OsMPK6 and OsMPK7 (Table 5). Figure S3 shows the top docking positions of OsMKK6-OsMPKs complexes.

**Table 5:** ZDOCK and RDOCK score of OsMKK6 against each of eleven rice MAPKs.

Protein Pair	Pose no.	ZDOCK Score	ZRANK Score	E_RDOCK Score	Clash
OsMPK20-2 & OsMKK6	91	13.44	−74.03	−**47.38**	0
OsMPK16-1 & OsMKK6	44	14.86	−79.41	−**36.63**	0
OsMPK21-2 & OsMKK6	32	16.02	−83.41	−**36.24**	0
OsMPK20-5 & OsMKK6	303	19.84	−48.6	−**27.43**	0
OsMPK6 & OsMKK6	5	16.44	−94.14	−**27.42**	0
OsMPK7 & OsMKK6	55	14.1	−75.08	−**24.06**	0
OsMPK20-3 & OsMKK6	3	19.4	−102.67	−23.4	0
OsMPK17-1 & OsMKK6	46	14.24	−78.1	−22.63	0
OsMPK4 & OsMKK6	7	15.56	−90.4	−20.78	0
OsMPK14 & OsMKK6	74	16.04	−68.73	−20.73	0
OsMPK3 & OsMKK6	129	16	−59.44	−19.81	0

E_R – Energy RDOCK score. Lower values of ZRANK score and E_RDOCK and higher ZDOCK scodre indicate top/better docking of the complex. Clash ‘0′ indicates no stearic clash between the proteins after refinement by RDOCK protocol.

In case of OsMKK10-2, after the initial docking (ZDOCK), OsMKK10-2 could not yield successful RDOCK with any of the OsMAPKs. Nonetheless, in the absence of E_RDOCK score, the other next important ‘ZRANK score’ [30] obtained from initial docking studies (ZDOCK) was used to predict the interaction of OsMKK10-2 with OsMAPKs (Table 6). Similar to E_RDOCK score, lower values of ZRANK score indicate better docking pair and thus can be used to predict top interacting pair of proteins. Following this approach, the top putative OsMKK10-2 interacting OsMAPKs were OsMPK7, OsMPK21-2, OsMPK20-3, OsMPK20-2, OsMPK16-1 and OsMPK6.

**Table 6:** ZDOCK score of OsMKK10-2 against each of eleven rice MAPKs.

Protein Pair	Pose no.	ZDOCK Score	ZRANK Score
OsMPK7 & OsMKK10-2	1	17.9	−**133.19**
OsMPK21-2 & OsMKK10-2	1	21.06	−**129.23**
OsMPK20-3 & OsMKK10-2	1	14.5	−**127.7**
OsMPK20-2 & OsMKK10-2	1	15.66	−**122.37**
OsMPK16-1 & OsMKK10-2	1	16.94	−**120.47**
OsMPK6 & OsMKK10-2	1	16.92	−**118.07**
OsMPK14 & OsMKK10-2	1	17.42	−116.88
OsMPK20-5 & OsMKK10-2	1	19.12	−116.17
OsMPK17-1 & OsMKK10-2	1	19.72	−108.47
OsMPK3 & OsMKK10-2	1	19.18	−108.38
OsMPK4 & OsMKK10-2	1	17.26	−107.59

Lower values of ZRANK score and higher ZDOCK score indicate top/better docking pose of the complex.

### Study of Interactions between Rice MAPKKs and MAPKs using Yeast Two-hybrid (Y2H) Assay

In Y2H analysis a total of 11 interactions were identified from 75 combinations of five MAPKKs and fifteen MAPKs. Each MAPKK was found to have at least one MAPK as interacting partner whereas, interestingly six MAPKs (OsMPK16-2, OsMPK17-1, OsMPK17-2 and OsMPK20-2, OsMPK20-5 and OsMPK21-2) were found not to be interacting with any of the MAPKKs. On the other hand, two MAPKs, OsMPK4 and OsMPK7 were found to have two distinct upstream MAPKKs as interacting proteins. Further, another MAPKK, OsMKK6 was found to interact with five different MAPKs.

For initial screening MAPKKs were fused with GAL4 DNA binding domain (vector pGBKT7, BD biosciences) while MAPKs with the GAL4 activation domain (vector pGADT7, BD biosciences). However, in case of OsMKK1, OsMKK1-pGBKT7 showed activation of reporter genes even in the presence of blank vector pGADT7 (Figure S4). Owing to this autoactivation property of OsMKK1, it was cloned in pGADT7, while MAPKs in pGBKT7 for checking OsMKK1-MAPK interactions. For the rest of the Y2H screenings MAPKKs and MAPKs cloned in pGBKT7 and pGADT7, respectively were used. The interactions observed in initial screens were later confirmed by repeating the experiments as well as by swapping the vectors, except for OsMKK1-MAPK interactions.

**Figure 4:**
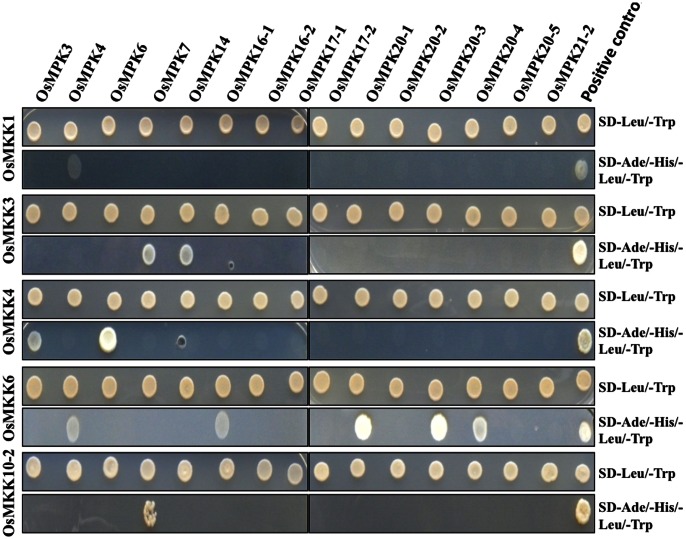
Yeast two-hybrid assay involving rice MAPKKs and MAPKs. OsMKK3, OsMKK4, OsMKK6 and OsMKK10-2 were fused with GAL4 DNA-binding domain and were used as a bait against each of the fifteen rice MAPKs (OsMPK3, OsMPK4, OsMPK6, OsMPK7, OsMPK14, OsMPK16-1, OsMPK16-2, OsMPK17-1, OsMPK17-2, OsMPK20-1, OsMPK20-2, OsMPK20-3, OsMPK20-4, OsMPK20-5 and OsMPK21-2) fused with GAL4 activation domain as preys. OsMKK1 fused with GAL4 activation domain as prey and fifteen MAPKs fused to DNA-binding domain baits were used to study protein interactions. Plasmids for bait and prey were co-transformed in AH109 yeast strain in different combinations as mentioned and selected on nutrient medium lacking Leu and Trp (upper panels). Interaction of bait and prey combinations were checked by assessing growth of co-transformed colonies streaked on selective medium lacking Ade, His, Leu and Trp (lower panels). p53 and SV40 large T-antigen are two proteins that are known to interact in yeast were used as a positive control.

OsMKK1, a group A MAPKK showed interaction with OsMPK4, a group B MAPK (Figure 4, panel 1-2). OsMKK6, the other member of group A MAPKK showed interaction with five MAPKs namely OsMPK4, OsMPK16-1, OsMPK20-1, OsMPK20-3 and OsMPK20-4 (Figure 4, panel 7-8).

OsMKK3 which alone constitutes group B rice MAPKK interacted with OsMPK7 and OsMPK14. Interestingly both OsMPK7 and OsMPK14 belonged to the same group of MAPKs (group C) (Figure 4, panel 3-4). However both the interactions were moderate in nature in comparison to the positive control.

A representative member of group C MAPKKs, OsMKK4 was found to interact with OsMPK3 and OsMPK6 (Figure 4, panel 5-6). The OsMKK4-OsMPK6 interaction was found to be stronger than the OsMKK4-OsMPK3 interaction.

OsMKK10-2, the only member of group D MAPKK which shows active transcription (data not shown) showed interaction with OsMPK7, albeit weak in nature (Figure 4, panel 9-10).

### Harmony between *in-silico* Predicted Protein-protein Interactions by Docking Study and Yeast Two-hybrid Screen

In order to substantiate the prediction of protein-protein interaction by computational docking, a vis-à-vis comparison of the same was made with Y2H screen (Table 7). In case of OsMKK3, the interacting MAPKs, OsMPK14 and OsMPK7 identified by Y2H were also present in the top six possible interactions and were found to be at 5^th^ and 6^th^ positions, respectively. For OsMKK4, two interactions identified by Y2H analyses OsMKK4-OsMPK6 and OsMKK4-OsMPK3 were also found to be at the top six possible interacting pairs and were at 3^rd^ and 5^th^ positions, respectively. Although, OsMKK5 was not included in the Y2H screening, a previous study demonstrated that OsMKK5 could activate OsMPK3 and OsMPK6 [13]. Both OsMPK3 and OsMPK6 were among the top six predicted interacting MAPKs of OsMKK5. In Y2H screen, OsMKK6 was shown to be interacting with five MAPKs namely, OsMPK4, OsMPK16-1, OsMPK20-1, OsMPK20-3, and OsMPK20-4. Since, OsMPK20-1 and OsMPK20-4 were not included in the docking study, only OsMPK16-1 could make to the list of top six predictions by computational dockings. In case of OsMKK10-2 the RDOCK protocol could not be successfully operated. Hence the second best preferred score, ZRANK was considered for prediction of possible interacting partner of OsMKK10-2. In Y2H screens OsMKK10-2 was shown to interact with OsMPK7, and interestingly the same was also the top most possible interacting MAPK in the computational docking studies.

**Table 7:** Direct comparison of yeast two-hybrid assay and computational dockings with respect to rice MAPKK-MAPK interaction.

MAPKK	Top six interacting MAPKs predicted by docking study	Interacting MAPKs identified by yeast two-hybrid screen
OsMKK1	NA	OsMPK4
OsMKK3	OsMPK20-3, OsMPK21-2, OsMPK20-2, OsMPK20-5, **OsMPK14** and **OsMPK7**	**OsMPK14** and **OsMPK7**
OsMKK4	OsMPK20-3, OsMPK21-2, **OsMPK6**, OsMPK20-5, **OsMPK3** and OsMPK16-1	**OsMPK6** and **OsMPK3**
OsMKK5	OsMPK20-5, **OsMPK3**, OsMPK21-2, OsMPK17-1, OsMPK20-3 and **OsMPK6**	NA
OsMKK6	OsMPK20-2, **OsMPK16-1**, OsMPK21-2, OsMPK20-5, OsMPK6 and OsMPK7	OsMPK4, **OsMPK16-1,** OsMPK20-1*, OsMPK20-3 and OsMPK20-4*
OsMKK10-2	**OsMPK7**, OsMPK21-2, OsMPK20-3, OsMPK20-2, OsMPK16-1 and OsMPK6	**OsMPK7**

MAPKs in bold font suggests that their interaction with respective MAPKK have been reported using both *in-silico* prediction and Y2H screen.

* MAPKs were not included in *in-silico* docking studies.

Over all, with the exception of OsMKK6, all the other MAPKK-MAPK interactions identified by Y2H study as well as those reported elsewhere were among the topmost interactions predicted by present computational docking. These results indicate that the computational docking approach using ZDOCK and RDOCK programmes are reliable and can be used for identification of possible protein-protein interactions.

## Discussion

The *in-silico* approach was used to predict protein-protein interaction between rice MAPKKs and MAPKs. The computational protein-protein docking, used in the present study can be utilized to predict large scale protein-protein interactions, especially for kinases. In the present study, this approach was followed in order to answer the following questions: 1) if the homology based 3-D structures of proteins could be used for protein–protein docking in the absence of experimentally elucidated structure? 2) If the computational protein-protein docking approach be employed to predict protein-protein interactions? If yes, 3) how reliable such an approach could be?

Since there are very limited reports which use the *in-silico* approach to study protein-protein interactions, it is considered necessary to render experimental basis to the outcomes of the docking studies. To achieve the same, computational protein-protein docking was further confirmed by directed Y2H analyses in the present study. The homology modelling approach was used to design 3D structures of MAPKK and MAPKs. Such homology models have been successfully used by earlier workers for docking studies [19], [31]. For protein-protein docking ZDOCK and RDOCK programs were used. These programs have successfully been used to recapitulat the structures of many known protein-protein complexes and have produced highly accurate predictions for multiple protein-protein targets in the CAPRI (Critical Assessment of Predicted Interactions) meetings [32], [33]. Further, to check the reliability of such predictions, Y2H screen was employed. Except for OsMKK6, all the other MAPKK interacting MAPKs identified by Y2H analyses were also found to be among the top MAPKK-MAPK interactions predicted by *in-silico* docking. Additionally, in case of OsMKK5, for which Y2H analysis could not be performed, the available literature furnishes evidence for the same. OsMKK5 was shown to activate OsMPK3 and OsMPK6 in-vitro [13] and both of the proteins have been predicted as interacting partners of OsMKK5.

Some of the the MAPKK-MAPK interactions identified by Y2H screens were not among the top most predictions by computational dockings. This could be due to inherent limitations of the use of homology models for docking or the docking process *per se*. Indeed the performance of the docking is often reduced when homology modelled proteins are employed, owing to the uncertainty of the models adding to the intrinsic noise of the docking results [34]. Moreover, the lack of knowledge of the structural motifs of MAPK or MAPKK that are involved in the interactions, limits the improvement. However, it is believed that there is no benchmark for studying the use of 3D models in protein-protein docking [34]. Therefore, it is important to have binding interface of modelled protein in near native conformation for precise prediction of protein–protein interactions.

In the present stuy this approach could be used to narrow down the number of interacting partners for protein in question from an array of proteins. It is also plausible that the MAPKK-MAPK interactions which were not identified by Y2H screen, however listed in the top possible interactions by computational docking could be genuine interactions and the Y2H system might have failed to identify the same. Indeed in case of Arabidopsis the directed Y2H screen identified only 23 MAPKK-MAPK interactions whereas protein microarrays could identify 48 MAPKK-MAPK interactions (MAPKK-MAPK phosphorylation network) [7], [8]. Interestingly, ‘OsMKK6-OsMPK6 interaction’ which was predicted by *in-silico* docking could not be identified in Y2H screen has recently been reported by phosphorylation assay [14]. In this perspective a further validation can be sought from a recent study [15] which showed PPI between rice MAPKKs and a few MAPKs. Singh et al [15] reported a total of 9 MAPK-MAPKK interactions comprising of three MAPKs and six MAPKKs (excluding OsWNK1, a With No Lysine Kinase). This information was used further to test the present *in-silico* protein-protein interaction predicted in the resent study (Table S2). From the 30 possible interactions for five MAPKKs (OsMKK3, OsMKK4, OsMKK5, OsMKK6 and OsMKK10-2) used in the present *in-silico* analysis, correct predictions could be made for 5 out of the 7 interactions reported by Singh et al. [15]. In addition to the current Y2H analyses, the interactions reported by Singh et al. [15] provide a valuable *in-vitro* and *in-vivo* validations for a few *in-silico* PPI predictions.

The prediction of interactions between rice MAPKKs and MAPKs by present *in-silico* approach with good accuracy clearly suggest that this approach holds great promise in prediction of protein-protein interactions. Further, the accuracy of these predictions suggests that 3D structures of proteins generated by homology modelling could be used for docking studies, provided that the binding sites are well conserved. If the binding interface of the modelled protein is in its near native conformation then the protein–protein interactions can be predicted more precisely. It is therfore, not necessary to find the whole length of protein in its native conformation to proceed with protein-protein docking, which makes homology models suitable for prediction of protein-protein interaction.

### Rice MAPKK-MAPK Interaction Network as Observed by Yeast Two-hybrid Screen

**Figure 5:**
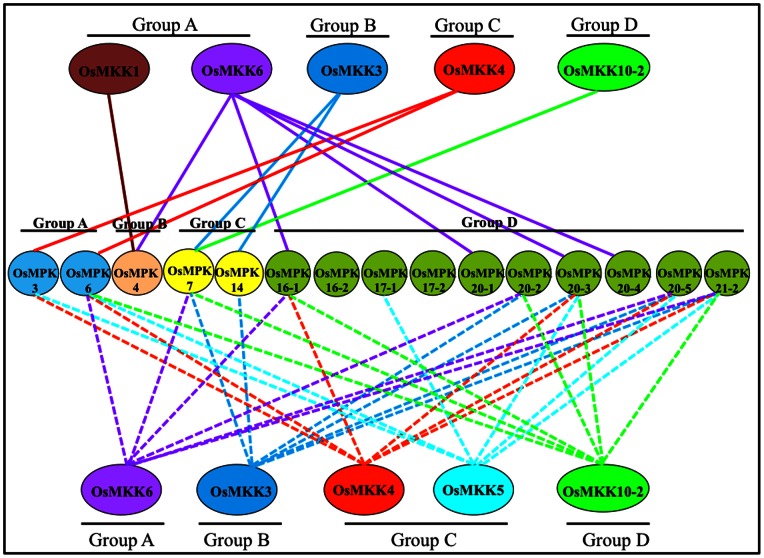
Protein-protein interaction network among rice MAPKKs and MAPKs based on Y2H analyses and *in-silico* predictions. Solid lines from upper panel OsMAPKKs to middle panel OsMAPKs indicate findings from yeast two-hybrid screen while dashed line from lower panel OsMAPKKs to middle panel OsMAPKs indicate findings from *in-silico* protein-protein dockings analyses. Lines originating from specific OsMAPKK are represented in same colour.

Our Y2H analyses provides a comprehensive MAPKK-MAPK interaction network in rice comprising of fifteen MAPKs and five MAPKKs across the groups A-D [5] (Figure 5). In similar direction, a recent study [15] showed upstream and downstream protein-protein interactions for four rice MAPKs, OsMPK6, OsMPK3, OsMPK4 and OsMPK20-4 (named as OsMPK1, OsMPK5, OsMPK6, and OsMPK8, respectively by Singh et al. [15]). We followed the nomeclature of MAPK cascade members according to Hamel et al. [5]. A proteome-wide Y2H screen of the rice leaf cDNA library identified 37, 10, 5, and 7 non redundant interactors for OsMPK6, OsMPK3, OsMPK4, and OsMPK20-4, respectively. However, only limited information is obtained from the study with respect to MAPKK-MAPK interaction network. Remarkably, there has not been any interacting MAPKKs reported for group C and group D (OsMPK20-4 interacted with WNK1 and no known MAPKK) members of rice MAPKs which together constitute 80% of the total MAPKs (twelve of a total of fifteen) in rice [5]. Further screening of cDNA library for identification of interacting proteins gives information for only those genes which are expressed at the given set of condition, developmental stage and tissue type and thus are likely to miss large set of interacting proteins. Therefore, directed protein-protein interaction screen would give a more elaborate understanding of the interactome. Nevertheless, the study [15] provides a significant insight into protein-protein interactions for selected MAPKs with the virtue of its elaborate *in-vitro* as well as *in-planta* validations. Therefore experimental evidences from Singh et al. [15] and other available reports (13,14), have also been considered for validation of *in-silico* predicted protein-protein interaction in the present study (Table S2).

OsMKK1, a member of group A MAPKK showed interaction only with OsMPK4. This interaction also has been reported along with an additional interacting pair OsMKK1-OsMPK6 [15]. Arabidopsis ortholog of OsMKK1, AtMKK1 interacted with AtMPK4 and AtMPK11 in yeast [7]. However, protein microarray, showed five interacting partners for AtMKK1 namely, AtMPK1, AtMPK2, AtMPK4, AtMPK5 and AtMPK6 [8]. AtMKK1 (AtMEK1) and AtMPK4 interaction has also been demonstrated in one of the earlier studies of MKK-MPK relationship [35].

OsMKK6 the other member of group A MAPKK, was found to interact with the maximum number of MAPKs (OsMPK4, OsMPK16-1, OsMPK20-1, OsMPK20-3 and OsMPK20-4). Among the identified interactions OsMKK6-OsMPK4 interaction has also been reported earlier [12] using Y2H system as well as the BiFC system. Other OsMKK6-OsMAPKs interactions have not been reported earlier. Although, OsMKK6-OsMPK3 and OsMKK6-OsMPK6 interactions have been reported earlier using Y2H studies/phosphorylation [11], [12], [14], in the present Y2H screen we could not obtain these interactions. Interestingly, another study using Y2H showed OsMKK6 (OsMEK1) interacting with OsMPK6 (OsMPK1) and OsMPK4 (OsMPK6) [15]. The inherent limitation of Y2H assay in producing both false positive and false negatives results may be accounted for such discrepancies. Further, it is also believed that different Y2H formats can also identify different protein-protein interactions [7]. The differenece in the Y2H format used in the above mentioned studies in comparision to that used in the present work could attribute to the failure to demonstrate a few known interactions. Another reason could be the transient nature of protein-protein interaction especially in the case of the signalling molecules. Additionally, it is interesting to note that AtMPK3, an ortholog of OsMPK3 in Arabidopsis showed no interaction with AtMKK6, but could interact with only one MAPKK, AtMKK4 [7]. Moreover, this interaction was also among the weakest interactions observed in the study.

OsMKK3, the only member of group B MAPKK, was found to interact with two rice MAPKs (OsMPK7 and OsMPK14) both belonging to group C of MAPKs [5]. However, another study has demonstrated the interaction of OsMKK3 (OsMEK8a) with OsMPK6 (OsMPK1), though the interaction was found to be weak in nature [15]. Further, two independent studies showed interaction of AtMKK3 (OsMKK3 ortholog in Arabidopsis) with group C members of Arabidopsis MAPKs (AtMPK1, AtMPK2, AtMPK7 and AtMPK14) [7], [36].

OsMKK4, a representative of group C MAPKK showed interaction with two MAPKs, OsMPK3 and OsMPK6. This is in agreement with another study where OsMKK4 was shown to activate OsMPK3 and OsMPK6 *in-vivo* and involved in phytoalexin biosynthesis in rice cell culture [13]. An independent study also reported similar interacting partner for OsMKK4 [15].

OsMKK10-2, the only member of group D MAPKKs, showed interaction with OsMPK7 albeit weak in nature. In contrast to this, in a previous study, OsMKK10-2 (OsMEK3) was found to interact with OsMPK6 (OsMPK1) [15]. Intriguingly, OsMKK10-2 showed only partial MAPKK consensus motif. The other paralogs, OsMKK10-1 and OsMKK10-3 also lack MAPKK consensus motif and showed no transcripts in any of the libraries of MPSS database (Data not shown). Further, the Arabidopsis AtMKK10 also lacks part of MAPKK consensus motif and is thought to be biologically non-functional [5], [37]. Y2H screen of AtMKK1 showed interaction only with one MAPK, AtMPK17 [7]. However, protein microarray revealed activation of five MAPKs including, AtMPK1, AtMPK2, AtMPK4, AtMPK5 and AtMPK6 [8].

Findings from the Y2H work showed that none of the rice MAPKKs included in the Y2H screen could interact with OsMPK16-2, OsMPK17-1, OsMPK17-2, OsMPK20-2, OsMPK20-5 and OsMPK21-1. Similarly, a Y2H study [7] comprising seven Arabidopsis MAPKs (AtMPK5, 8, 9, 12, 16, 18, and 19) showed no interactions with any of the selected AtMAPKKs. In order to obtain a substatial interaction of MAPKKs with MAPKs, we might need scaffold proteins as observed in yeast. Scaffold proteins bring MAPK components together to enhance specificity and accelerate their activation and reaction rates. For example, yeast Ste5 was found to interact with an upstream G-protein and along with all three components of the MAPK cascade [38].

Comparison of MAPKKs and MAPKs orthologs between rice and Arabidopsis revealed a few new interacting modules. Changes in docking domain over the period of evolution could be accounted for these new interactions since docking domains are major determinants of the specificity of interactions between MAPKKs and MAPKs [39]–[41].

The present work apart from improving our understanding about the MAPK-MAPKK interaction network in rice, also shows that the computational approach could be employed to explore large scale protein-protein interactions.

### Conclusions

The MAPK and MAPKK interaction network is crucial to understand the MAPK signalling pathways at cellular level. The present work represents an *in-silico* docking approach to predict protein-protein interactions between rice MAPKKs and MAPKs. To achieve the same, 3D structures of eleven MAPKs and five MAPKKs were predicted by homology modelling. ZDOCK and RDOCK docking programmes were used for computational dockings and top possible MAPKK-MAPK interacting pairs were predicted. Further to confirm the reliability of this approach, Y2H analysis was performed for conforming MAPKK-MAPK interactions. Except for one MAPKK, all the other interacting partners identified in Y2H assay were listed in the top possible interactions by computational dockings. The results suggest that 3D structure built by homology modelling could be used for docking studies. Since the top interacting pairs identified by docking in the present work could not be confirmed by Y2H assay in each case, this approach may be suitable mainly for narrowing down the interacting partners from several proteins to a fewer members. The protein-protein interaction study of MAPKKs and MAPKs gives a comprehensive interaction network for rice MAPKK and MAPK.

## Supporting Information

Figure S1 **Top docking poses of OsMKK4 with rice MAPKs.** The best docking positions of OsMKK4 with each of the eleven rice MAPKs (OsMPK3, OsMPK4, OsMPK6, OsMPK7, OsMPK14, OsMPK16-1, OsMPK17-1, OsMPK20-2, OsMPK20-3, OsMPK20-5 and OsMPK21-2) are shown. OsMKK4 is represented as a wireframe pattern whereas all OsMAPKs as a solid ribbon form.
(PDF)

Figure S2 **Top docking poses of OsMKK5 with rice MAPKs.** The best docking positions of OsMKK5 with each of the eleven rice MAPKs (OsMPK3, OsMPK4, OsMPK6, OsMPK7, OsMPK14, OsMPK16-1, OsMPK17-1, OsMPK20-2, OsMPK20-3, OsMPK20-5 and OsMPK21-2) are shown. OsMKK5 is represented as a wireframe pattern whereas all OsMAPKs as a solid ribbon form.
(PDF)

Figure S3 **Top docking poses of OsMKK6 with rice MAPKs.** The best docking positions of OsMKK6 with each of the eleven rice MAPKs (OsMPK3, OsMPK4, OsMPK6, OsMPK7, OsMPK14, OsMPK16-1, OsMPK17-1, OsMPK20-2, OsMPK20-3, OsMPK20-5 and OsMPK21-2) are shown. OsMKK6 is represented as a wireframe pattern whereas all OsMAPKs as a solid ribbon form.
(PDF)

Figure S4 **Yeast two-hybrid assay control experiment.** *OsMKK1, OsMKK3, OsMKK4, OsMKK6* and *OsMKK10-2* cloned in pGBKT7 were co-transformed with blank pGADT7 to AH109. The co-transformants were selected on double drop out medium and later patched on quadruple drop out medium to check auto-activation of the reporter genes.
(PDF)

Table S1 **Details of the proteins used as templates for homology modelling of rice MAPKKs and MAPKs.**
(PDF)

Table S2 **Rice MAPK and MAPKKs interactions predicted by** ***in-silico*** **docking and its validation as observed in the experimental evidences from current Y2H analyses and available literatures.**
(PDF)
