# Supplementary material for: Rice Mitogen Activated Protein Kinase Kinase and Mitogen Activated Protein Kinase Interaction Network Revealed by In-Silico Docking and Yeast Two-Hybrid Approaches
Source: PLoS One. 2013 May 30;8(5):e65011. doi: 10.1371/journal.pone.0065011 (PMC3667834; doi:10.1371/journal.pone.0065011)
Supplement: Figure S2 — Top docking poses of OsMKK5 with rice MAPKs. The best docking positions of OsMKK5 with each of the eleven rice MAPKs (OsMPK3, OsMPK4, OsMPK6, OsMPK7, OsMPK14, OsMPK16-1, OsMPK17-1, OsMPK20-2, OsMPK20-3, OsMPK20-5 and OsMPK21-2) are shown. OsMKK5 is represented as a wireframe pattern whereas all OsMAPKs as a solid ribbon form. (PDF) [file pone.0065011.s002.pdf]

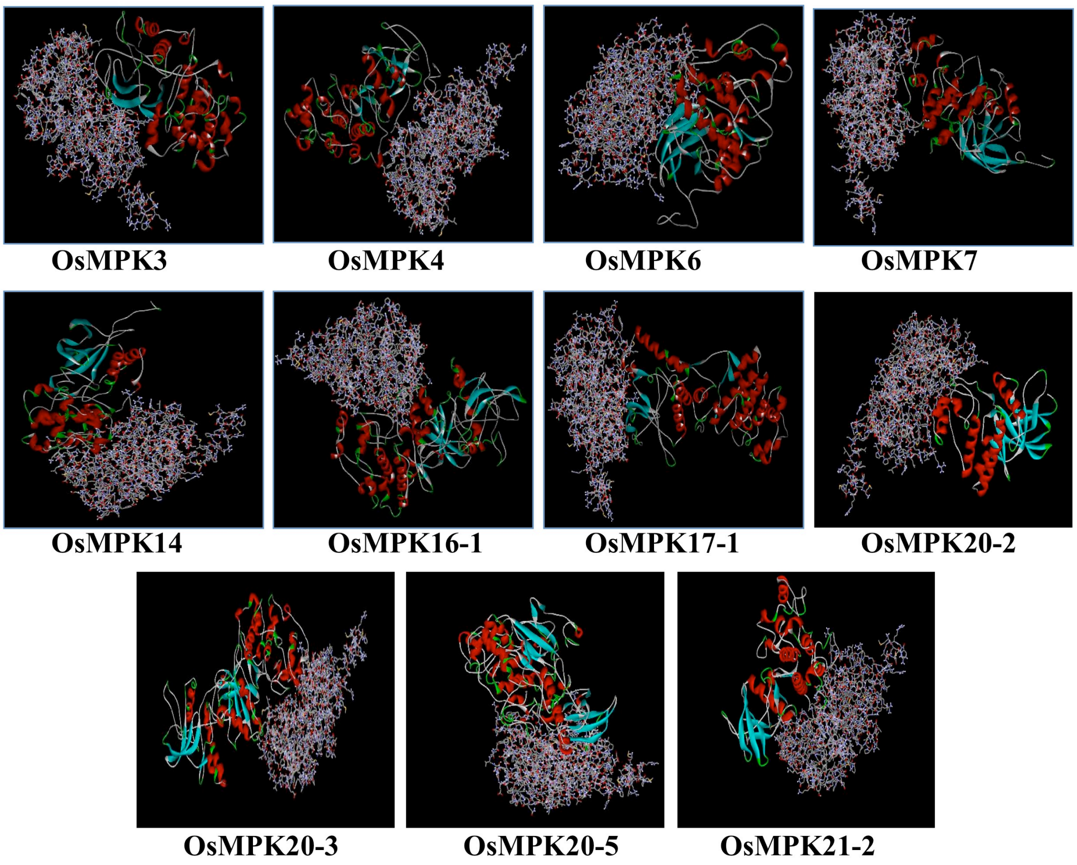

Figure S2. Top docking poses of OsMKK5 with rice MAPKs. The best docking positions OsMKK5 with each of the eleven rice MAPKs (OsMPK3, OsMPK4, OsMPK6, OsMPK7, OsMPK14, OsMPK16-1, OsMPK17-1, OsMPK20-2, OsMPK20-3, OsMPK20-5 and OsMPK21-2) are shown. OsMKK5 is represented as a wireframe pattern whereas all OsMAPKs as solid ribbon form.
