# Supplementary material for: Rice Mitogen Activated Protein Kinase Kinase and Mitogen Activated Protein Kinase Interaction Network Revealed by In-Silico Docking and Yeast Two-Hybrid Approaches
Source: PLoS One. 2013 May 30;8(5):e65011. doi: 10.1371/journal.pone.0065011 (PMC3667834; doi:10.1371/journal.pone.0065011)
Supplement: Figure S4 — Yeast two-hybrid assay control experiment. OsMKK1, OsMKK3, OsMKK4, OsMKK6 and OsMKK10-2 cloned in pGBKT7 were co-transformed with blank pGADT7 to AH109. The co-transformants were selected on double drop out medium and later patched on quadruple drop out medium to check auto-activation of the reporter genes. (PDF) [file pone.0065011.s004.pdf]

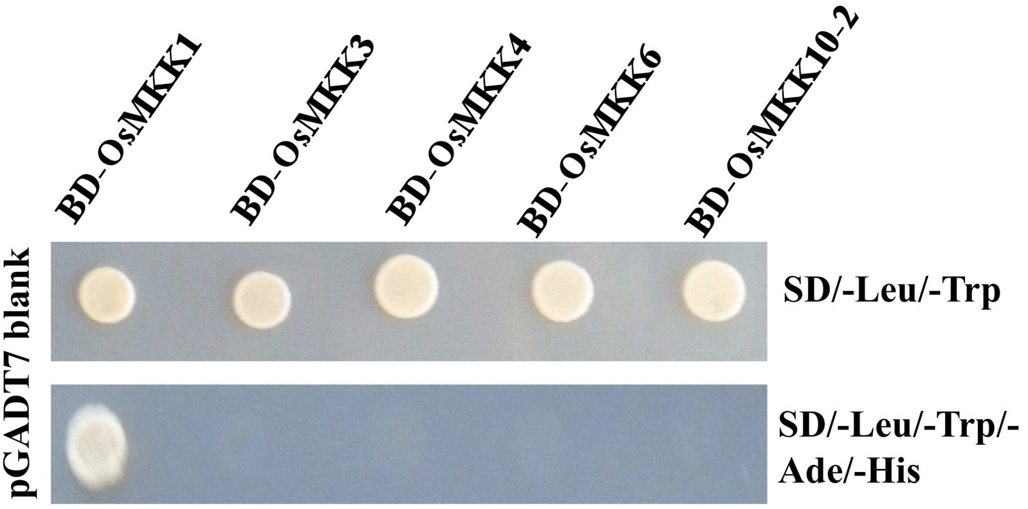

Figure S4. Yeast two-hybrid assay control experiment. *OsMKK1, OsMKK3, OsMKK4, OsMKK6* and *OsMKK10-2* cloned in pGBKT7 were co-transformed with blank pGADT7 to AH109. The co-transformants were selected on double drop out medium and later patched on quadruple drop out medium to check auto-activation of reporter genes.
