# Supplementary material for: Rice Mitogen Activated Protein Kinase Kinase and Mitogen Activated Protein Kinase Interaction Network Revealed by In-Silico Docking and Yeast Two-Hybrid Approaches
Source: PLoS One. 2013 May 30;8(5):e65011. doi: 10.1371/journal.pone.0065011 (PMC3667834; doi:10.1371/journal.pone.0065011)
Supplement: Table S1 — Details of the proteins used as templates for homology modelling of rice MAPKKs and MAPKs. (PDF) [file pone.0065011.s005.pdf]

Table S1. Details of proteins used as templates for homology modelling of rice MAPKKs and MAPKs.

Protein name	PDB Id	Molecule name	Source organism
OsMPK3	1BMK	Mitogen activated protein kinase P38	*H. sapiens*
	2ZOQ	Mitogen activated protein kinase 3	*H. sapiens*
OsMPK4	2B9H	Mitogen activated protein kinase FUS3	*S. cerevisiae*
	3MPT_A	Mitogen-activated protein kinase 14 (p38 kinase)	*H. sapiens*
	1WZY_A	Mitogen-activated protein kinase 1	*H. sapiens*
OsMPK6	2GPH_A	Mitogen-activated protein kinase 1	*Rattus norvegicus*
	2OJG	Mitogen-activated protein kinase 1	*H. sapiens*
	3DTI	IRRE protein	*Deinococcus deserti*
OsMPK7	2GPH_A	Mitogen-activated protein kinase 1	*R. norvegicus*
	ITVO_A	ERK2 in complex with small molecular inhibitor	*H. sapiens*
	3GC9_A	Mitogen-activated protein kinase 11	*H. sapiens*
OsMPK14	2FST_X	Mitogen-activated protein kinase 14	*H. sapiens*
	3COI_A	Mitogen-activated protein kinase 13	*H. sapiens*
	3MPT_A	Mitogen-activated protein kinase 14	*H. sapiens*
OsMPK16-1	2GPH_A	Mitogen-activated protein kinase 1	*R. norvegicus*
	1WZY_A	Mitogen-activated protein kinase 1	*H. sapiens*
	2ZOQ_A	Mitogen-activated protein kinase 3	*H. sapiens*
OsMPK17-1	2GPH_A	Mitogen-activated protein kinase 1	*R. norvegicus*
	2ZOQ_A	Mitogen-activated protein kinase 3	*H. sapiens*
	3FI4_A	Mitogen-activated protein kinase 14	*H. sapiens*
OsMPK20-2	1WZY_A	Mitogen-activated protein kinase 1	*H. sapiens*
	2B9F_A	Mitogen-activated protein kinase FUS3	*S. cerevisiae*
	3HVC	Mitogen-activated protein kinase 14	*H. sapiens*
	3GP0_A	Mitogen-activated protein kinase 11	*H. sapiens*
OsMPK20-3	2OJG_A	Mitogen-activated protein kinase 1	*H. sapiens*
	2ZOQ_A	Mitogen-activated protein kinase 3	*H. sapiens*
	2F9G_A	Mitogen-activated protein kinase FUS3	*S. cerevisiae*
	2GPH_A	Mitogen-activated protein kinase 1	*R. norvegicus*
OsMPK20-5	2OJG_A	Mitogen-activated protein kinase 1	*H. sapiens*
	2ZOQ_A	Mitogen-activated protein kinase 3	*H. sapiens*
	2GPH_A	Mitogen-activated protein kinase 1	*R. norvegicus*
OsMPK21-2	2GPH_A	Mitogen-activated protein kinase 1	*R. norvegicus*
	3GCU_A	Mitogen-activated protein kinase 14	*H. sapiens*
	2Z7L_A	Mitogen-activated protein kinase 1	*R. norvegicus*

	2B9H	Mitogen-activated protein kinase FUS3	*S. cerevisiae*
OsMKK3	3EQC	Dual specificity mitogen-activated protein kinase kinase 1	*H. sapiens*
	3DV3_A	Dual specificity mitogen-activated protein kinase kinase 1	*H. sapiens*
	2JS1	Uncharacterized protein yvfG	*Bacillus subtilis*
OsMKK4	3EQC_A	Dual specificity mitogen-activated protein kinase kinase 1	*H. sapiens*
	2DYL_A	Dual specificity mitogen-activated protein kinase kinase 7	*H. sapiens*
	2W4O_A	calcium/calmodulin-dependent protein kinase type IV	*H. sapiens*
	1O6L	RAC-BETA Serine/Threonine protein kinase	*H. sapiens*
OsMKK5	3EQC	dual specificity mitogen-activated protein kinase kinase 1	*H. sapiens*
	2X4F_A	MYOSIN LIGHT CHAIN KINASE FAMILY MEMBER 4	*H. sapiens*
	2W4O_A	CALCIUM/CALMODULIN-DEPENDENT PROTEIN KINASE TYPE IV	*H. sapiens*
	1YHV_A	Serine/threonine-protein kinase PAK 1	*H. sapiens*
OsMKK6	3EQC_A	Dual specificity mitogen-activated protein kinase kinase 1	*H. sapiens*
	2VWI	SERINE/THREONINE-PROTEIN KINASE OSR1	*H. sapiens*
	1O6L_A	RAC-BETA SERINE/THREONINE PROTEIN KINASE	*H. sapiens*
OsMKK10-2	2J51_A	STE20-LIKE SERINE/THREONINE-PROTEIN KINASE	*H. sapiens*
	2F2U_A	Rho-associated protein kinase 2	*Bos taurus*
	2BMC_A	SERINE THREONINE-PROTEIN KINASE 6	*H. sapiens*
	3COM_A	Serine/threonine-protein kinase 4	*H. sapiens*
