# Supplementary material for: Rice Mitogen Activated Protein Kinase Kinase and Mitogen Activated Protein Kinase Interaction Network Revealed by In-Silico Docking and Yeast Two-Hybrid Approaches
Source: PLoS One. 2013 May 30;8(5):e65011. doi: 10.1371/journal.pone.0065011 (PMC3667834; doi:10.1371/journal.pone.0065011)
Supplement: Table S2 — Rice MAPK and MAPKKs interactions predicted by in-silico docking and its validation as observed in the experimental evidences from current Y2H analyses and available literatures. (PDF) [file pone.0065011.s006.pdf]

Table S2. Rice MAPK and MAPKKs interactions predicted by in-silico docking and its validation as observed in the experimental evidences from current Y2H analyses and available literature.

**In-silico PPI predictions from current study**		**Current Y2H study**	**Other reports**
OsMKK3 (OsMEK8a)	OsMPK20-3 (OsMPK11)	X	X (15)
	OsMPK21-2	X	NA
	OsMPK20-2	X	NA
	OsMPK20-5	X	NA
	OsMPK14 (OsMPK3)	√	X (15)
	OsMPK7 (OsMPK4)	√	X (15)
OsMKK4 (OsMEK6)	OsMPK20-3 (OsMPK11)	X	X (15)
	OsMPK21-2	X	NA
	OsMPK6 (OsMPK1)	√	√ (13,15)
	OsMPK20-5	X	NA
	OsMPK3 (OsMPK5)	√	√ (13,15)
	OsMPK16-1	X	NA
	OsMPK20-3 (OsMPK11)	X	X (15)
OsMKK5 (OsMEK7b)	OsMPK20-5	NA	NA
	OsMPK3 (OsMPK5)	NA	X (15); √(13)
	OsMPK17-1	NA	NA
	OsMPK21-2	NA	NA
	OsMPK20-3 (OsMPK11)	NA	X (15)
	OsMPK6 (OsMPK1)	NA	X(15); √(13)
OsMKK6 (OsMEK1)	OsMPK20-2	X	NA
	OsMPK16-1	√	NA
	OsMPK21-2	X	NA
	OsMPK20-5	X	NA
	OsMPK6 (OsMPK1)	X	√ (14,15)
	OsMPK7 (OsMPK4)	X	X(15)
OsMKK10-2 (OsMEK3)	OsMPK7 (OsMPK4)	√	X(15)
	OsMPK21-2	X	NA
	OsMPK20-3 (OsMPK11)	X	X(15)
	OsMPK20-2	X	NA
	OsMPK16-1	X	NA

The nomenclature of MAPKs and MAPKKs used in the present work is based on Hamel et al. [5] while the corresponding MAPKs and MAPKKs names followed by Singh et al. [15] are shown in parentheses. “X”: No interaction; “√”: Interatcion; “NA”; Not Available.
